# Ketogenic metabolic therapy for treatment-resistant post-traumatic stress disorder (PTSD): a retrospective case report

**DOI:** 10.3389/fnut.2026.1755107

**Published:** 2026-02-17

**Authors:** Nicole Laurent

**Affiliations:** Family Renewal, Inc., Vancouver, WA, United States

**Keywords:** case report, ketogenic diet, ketogenic metabolic therapy, metabolic psychiatry, post-traumatic stress disorder, treatment-resistant, military sexual trauma, nutritional ketosis

## Abstract

**Background:**

This case report examines the application of ketogenic metabolic therapy (KMT), also known as the ketogenic diet, in a patient with treatment-resistant Post-Traumatic Stress Disorder (PTSD) resulting from military sexual trauma (MST) who was nonresponsive to conventional interventions including psychotherapy and pharmacotherapy. Metabolic dysfunction can contribute to persistent symptoms highlighting the need for novel treatment approaches.

**Methods:**

A retrospective analysis was conducted on a 45-year-old female patient who underwent a structured ketogenic dietary intervention for 25 weeks. Therapeutic carbohydrate restriction was initiated by the patient 9 days before formal treatment with rapid and early improvements in mood prior to working with the KMT professional. Clinical response was monitored using validated instruments, including Post-Traumatic Stress Disorder Checklist for Diagnostic and Statistical Manual of Mental Disorders (DSM)-5 (PCL-5), Patient Health Questionnaire-9 (PHQ-9), Generalized Anxiety Disorder-7 (GAD-7), Depression Anxiety Stress Scales-42 (DASS-42), as well as daily metabolic measurements assessing nutritional ketosis.

**Results:**

Quantitative assessments demonstrated significant clinical improvement. The PCL-5 score decreased from 32 at the intervention baseline to 2 at 27 weeks. The PHQ-9 score declined from 10 to 0 and the GAD-7 score decreased from 6 to 0. DASS-42 further confirmed the resolution of depressive, anxious, and stress symptoms. Qualitative data corroborated these findings, with the patient reporting enhanced mood stability, improved cognitive function, and a renewed sense of emotional wellbeing.

**Conclusion:**

Targeted KMT may be effective against the metabolic dysfunction underlying treatment-resistant PTSD. The consistent improvements across multiple psychometric assessments, supported by qualitative reports, warrant further controlled investigations into the clinical utility of this intervention.

## 1 Introduction

Post-Traumatic Stress Disorder (PTSD) can follow diverse trauma exposures including interpersonal violence in childhood or adulthood, intimate partner violence, nonsexual assault, serious accidents such as motor vehicle collisions, natural or human caused disasters, medical or surgical trauma, witnessing injury or death, sudden unexpected death of a close person, war and combat, forced displacement, and repeated or occupational exposure to traumatic events (1). PTSD diagnosis according to DSM-5 criteria requires trauma exposure (Criterion A), followed by symptom clusters including intrusive symptoms (Criterion B), avoidance of trauma-related stimuli (Criterion C), negative alterations in cognition and mood (Criterion D), and alterations in arousal and reactivity (Criterion E) (1). PTSD affects an estimated 3.4–26.9% of individuals in the United States, with prevalence varying based on trauma exposure and population characteristics (2, 3). Recent studies indicate that PTSD prevalence has been increasing, with rising cases linked to global conflicts, natural disasters, and societal stressors (3). Additionally, emerging research suggests a potential role of metabolic interventions in addressing psychiatric disorders, including PTSD, through mechanisms such as reduction in neuroinflammation, mitochondrial support and oxidative stress (4–7). Despite treatment advances an estimated 39.23% do not respond to first line psychological interventions, confirming the need for novel therapeutic options. Nonresponse rates vary widely across studies, ranging from 0 to 85.7%. Determinants of poor treatment outcomes include being male, older age, and refugee or veteran status. These findings confirm the need for alternative interventions to address persistent, disabling symptoms (8).

### 1.1 Current standard treatments

Psychotherapy and pharmacotherapy remain the primary treatment approaches for PTSD although both have significant limitations including high nonresponse rates, adverse effects and modest long-term efficacy (9–11). Trauma-focused psychotherapy such as Cognitive Behavioral Therapy (CBT) and Eye Movement Desensitization and Reprocessing (EMDR) are considered first line treatments (12, 13). Clinical guidelines recommend trauma-focused CBT, including cognitive processing therapy (CPT) and prolonged exposure (PE) as first line treatments and endorse EMDR, although there is less consensus regarding its superiority (12–14). Findings from a network meta-analysis confirm that trauma-focused CBT and EMDR effectively reduce PTSD symptoms, with EMDR demonstrating the strongest immediate effects and CPT showing the most sustained long-term benefits (14). However, studies directly comparing these interventions have found no significant differences in outcomes, reinforcing the need for individualized treatment selection (13, 15, 16).

Individuals who do not achieve symptom remission with psychotherapy alone are often placed on pharmacotherapy as an additional or alternative intervention (9, 10). A recent narrative review demonstrates the complexity of PTSD treatment, showing the mixed efficacy of both pharmacological and psychotherapeutic interventions and the ongoing challenges in achieving symptom remission (10). Selective serotonin reuptake inhibitors demonstrate modest symptom reductions, high nonresponse rates, and adverse effects, including weight gain, sexual dysfunction, emotional blunting, and agitation, reducing adherence (9, 11, 17–19). More recently, ketamine has demonstrated rapid PTSD symptom relief, but concerns about long-term efficacy, dissociative effects, and potential addiction limit broader adoption (20–22). Given the limitations of existing treatments, research has expanded beyond symptom management to understanding underlying biological mechanisms that may sustain PTSD (23).

### 1.2 The role of metabolism in PTSD

Metabolic dysfunction contributes to PTSD symptomatology through observed glucose hypometabolism in brain regions implicated in trauma responses (24, 25). PTSD is increasingly recognized as a systemic metabolic disorder, characterized not only by altered brain metabolism but also by broader disturbances in neuroendocrine function, sympathetic nervous system activity, inflammatory pathways and immune dysregulation (26–28). These metabolic disturbances likely affect neuronal function, neurotransmitter activity, synaptic transmission and neuronal plasticity, although the specific mechanistic contributions to PTSD symptomatology remain unclear (29, 30). Disruptions in neurotransmitter balance are compounded by impaired energy metabolism, which reduces the efficiency of synaptic transmission and neuronal plasticity (31, 32). Elevated glutamate and norepinephrine, along with reduced GABA levels, contribute to hyperarousal, anxiety, and intrusive memories (33). These neurotransmitter disruptions arise from metabolic dysregulation and mitochondrial impairment, processes that compromise neuronal function by disrupting oxidative phosphorylation. This disruption reduces ATP availability and increases oxidative stress, ultimately impairing neurotransmitter synthesis, storage, release, and neuronal signaling (31, 34–36).

Excessive oxidative stress has been implicated in the progressive loss of neuronal integrity, contributing to cognitive and emotional dysregulation (5, 37). Elevated levels of proinflammatory cytokines observed in PTSD may reflect sustained neuroimmune activation, potentially contributing to neurotoxic conditions implicated in cognitive and emotional dysfunction (38, 39). Elevated proinflammatory cytokines, including IL-6, TNF-α, and IL-1β, have been associated with increased symptom severity and cognitive impairments in PTSD (40, 41).

These metabolic dysfunctions, including glucose hypometabolism, neurotransmitter imbalance, oxidative stress, and neuroinflammation (6), form interrelated mechanisms underlying PTSD symptom persistence and severity. Targeting neurotransmitter imbalance, oxidative stress, and neuroinflammation represents a promising therapeutic avenue in PTSD treatment (4, 6). Given these overlapping mechanisms, the ketogenic diet presents a promising therapeutic strategy for PTSD, warranting clinical investigation.

### 1.3 The ketogenic diet as a metabolic intervention

KMT is a high-fat, low-carbohydrate dietary intervention that shifts energy metabolism from glucose utilization to ketone body production, primarily beta-hydroxybutyrate. Originally developed as an evidence-based therapy for epilepsy, KMT has since demonstrated efficacy across neurological disorders, including Alzheimer’s disease and migraine (42), as well as psychiatric conditions such as anxiety, depression, and other severe mental disorders (43–45). KMT directly targets metabolic pathways relevant to PTSD pathophysiology, improving mitochondrial function, reducing oxidative stress and neuroinflammation, and supporting neurotransmitter balance (7, 46, 47). At the biochemical level, KMT enhances mitochondrial function (7, 44), reduces neuroinflammation (7, 46), mitigates oxidative stress (7, 47), and supports neurotransmitter synthesis and function (34, 44), each highly relevant to PTSD symptomatology. Recent literature further positions KMT as a potential transdiagnostic therapy, emphasizing its pleiotropic effects across various neuropsychiatric conditions (4, 6).

A case series reports two adults diagnosed with PTSD and comorbid psychiatric conditions who achieved complete symptom remission using individualized KMT interventions (48). In Case Study 2, a 53-year-old male with PTSD, generalized anxiety disorder, and major depressive disorder experienced full remission of PTSD symptoms after 12 weeks on a KMT. Similarly, Case Study 3 was a 39-year-old female diagnosed with PTSD, generalized anxiety disorder, and panic disorder who achieved complete remission following a 10-week KMT protocol. A single retrospective case report described a 38-year-old female patient with treatment-resistant PTSD, ADHD, and binge-eating disorder who achieved full psychiatric symptom remission after a structured ketogenic dietary intervention lasting 12 weeks (49). These cases provide preliminary evidence supporting the feasibility and clinical relevance of KMT therapy for treatment-resistant PTSD.

A small feasibility study also explored the effects KMT supplemented with exogenous ketones in individuals with PTSD. Two of three participants reported symptom improvements following the four-week intervention, though the small sample size and short study duration limited generalizability of the study findings (50). While exogenous ketone supplementation is being investigated as a standalone or adjunctive psychiatric treatment, it remains unclear whether transient ketosis from supplementation sufficiently replicates the sustained metabolic adaptations associated with nutritional ketosis (51).

### 1.4 Case study introduction

This case report details the experience of an individual with treatment-resistant PTSD who achieved symptom remission following adherence to KMT. Despite extensive prior treatment, including multiple forms of psychotherapy and various pharmacological interventions, the individual continued to experience debilitating PTSD symptoms. Conventional approaches failed to provide lasting relief, prompting the exploration of alternative strategies. Following the implementation of KMT, the individual reported substantial reductions in PTSD symptom severity, improved emotional regulation, and enhanced overall functioning. This report suggests ketogenic dietary therapy as a potential intervention warranting further investigation in individuals with treatment-resistant PTSD.

## 2 Case presentation

### 2.1 Clinical background

The patient was a 45-year-old woman diagnosed with PTSD for approximately 2 years. Her psychiatric symptoms began in adolescence, initially presenting as anxiety-related physical complaints. Symptoms intensified significantly following multiple episodes of military sexual trauma (MST) occurring over a two-year period during her Air Force service. Raised in a highly religious environment, she experienced significant shame, guilt, and self-blame, which delayed trauma disclosure and exacerbated subsequent psychological symptoms, including chronic anxiety, frequent dissociative episodes, severe body dysmorphia, intimacy difficulties, and pronounced social isolation.

Additional trauma occurred during her 14 years in nursing from repeated exposure to emotionally traumatic clinical situations, particularly in acute care settings. These experiences significantly exacerbated her anxiety and paranoia, directly contributing to occupational instability characterized by frequent job changes and multiple medical leaves due to the inability to manage the intense emotional and psychological demands of environments. The patient increased her alcohol consumption shortly after being diagnosed with PTSD but quickly discontinued upon recognizing the associated risks.

Her treatment history included approximately 18 years of intermittent psychotherapy, initially centered on parenting support and subsequently focusing extensively on trauma processing. Prior therapeutic interventions included EMDR, CBT, IFS, drama therapy, peer-to-peer PTSD support incorporating meditation, and a psilocybin-assisted retreat. Additionally, she participated in therapeutic workshops and briefly attended residential PTSD treatment for 17 days, leaving prematurely due to adverse experiences including reported difficulties tolerating the emotional intensity and group-based format of the residential program. Assessments conducted during this residential treatment approximately 2 years prior to initiating ketogenic metabolic therapy (KMT) revealed severe PTSD and related psychiatric symptoms, evidenced by scores of 64 on the Post-Traumatic Stress Disorder Checklist for (DSM)-5 (PCL-5), 39 on the Beck Depression Inventory (BDI), and 45 on the Beck Anxiety Inventory (BAI), confirming significant symptom severity and poor response to prior interventions (Supplementary Table 1).

PTSD was formally diagnosed according to DSM-5 criteria based on repeated clinical evaluations conducted by multiple providers across different treatment contexts. Diagnostic reasoning was informed by the persistence of trauma-linked intrusive symptoms, avoidance, negative alterations in cognition and mood, and hyperarousal following military sexual trauma. Differential diagnoses considered included major depressive disorder and generalized anxiety disorder, which were insufficient to account for the trauma-specific symptom pattern and course. Diagnostic challenges included delayed disclosure of military sexual trauma, chronic symptom presentation over many years, and symptom overlap with mood and anxiety disorders. Medication trials included escitalopram, which initially provided relief but caused emotional numbness; sertraline, which was discontinued due to headaches and nausea; Wellbutrin, which was stopped following an allergic reaction; and Abilify, which was prescribed but not initiated as the patient was concerned about side effects. She tolerated nightly low-dose naltrexone 4.5 mg, reported minimal symptom relief and remained on this medication at KMT initiation.

Given the persistent limitations and inadequate relief provided by conventional psychotherapy and medication treatments, the patient proactively sought KMT after independently researching alternative treatment options online and learning about ketogenic dietary interventions for mental health. Motivated specifically by the ongoing severity of her psychiatric symptoms and ineffective symptom management through previous interventions, she chose KMT based on preliminary research into its potential psychiatric benefits.

Her treatment involved one clinician specifically trained in KMT, who worked independently from her primary care physician and existing mental health providers.

### 2.2 Ketogenic metabolic therapy intervention strategy

The patient reported minimal appetite for approximately 2 years before initiating KMT. During this period, she struggled to maintain a nutritionally adequate diet and selected foods based on immediate preference, often favoring carbohydrate-rich and processed options. At baseline prior to KMT initiation, weight was 142.7 lbs (64.7 kg) with a body mass index of 28.8 kg/m^2^ (overweight). Due to the inconsistency of her intake before diet initiation, comprehensive baseline dietary data were unavailable. She began restricting dietary carbohydrates 9 days before her initial KMT session, preventing a baseline dietary analysis of carbohydrate intake. The patient reported noticeable improvement within 5 days of dietary changes.

Professional support included an initial one-hour virtual consultation, followed by structured individual sessions of shorter duration. The patient engaged weekly to bi-weekly 30-min virtual sessions for approximately 7 weeks, then gradually transitioned to monthly intervals for 5 months. The patient was initiated on a 1.5:1 ratio KD, eating three meals a day. Macronutrient distribution was set at 77% fat (144 g), 18% protein (76 g), and 5% net carbohydrates (20 g), totaling approximately 1,680 calories per day.

Protein sources included organic pasture-raised eggs, chicken breakfast links, grass-fed beef snack sticks, sardines in olive oil, grass-fed steak, wild salmon, string cheese, and organic chicken patties. Primary dietary fat sources included coffee prepared with heavy whipping cream and medium chain triglycerides (MCT) oil, dry-roasted macadamia nuts with sea salt, pecan halves, grass-fed unsalted butter, avocado oil mayonnaise, organic pasture-raised eggs, avocados, and additional dairy sources such as cheese. Carbohydrates intake was minimal and included macadamia nuts, pecan halves, avocados, broccoli, and cucumbers. Treats consisted of high-protein, low-carb cereal bars sweetened with allulose and monk fruit.

**Figure 1:**
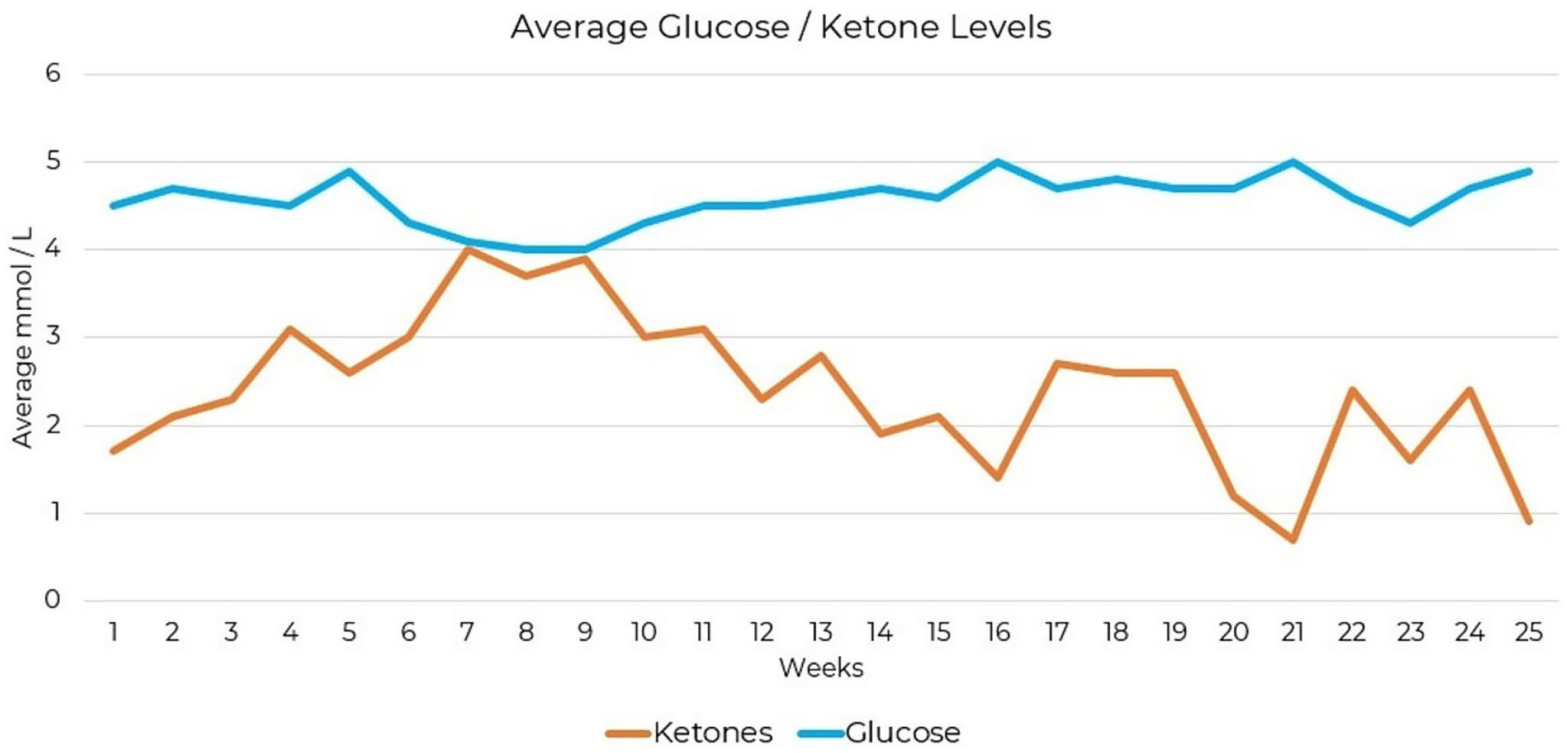
Average weekly glucose and ketone levels following over 25 weeks of ketogenic metabolic therapy (KMT).

Personalized supplementation for KMT was formulated based on suspected dietary insufficiencies identified through dietary intake assessment and included a methylated B-complex, vitamins A, C, D3, K, and E, and trace minerals including boron, chromium, iodine, magnesium, manganese, molybdenum, potassium, selenium, and zinc. Supplementation was initiated approximately 4 weeks after KMT initiation. Upon adopting a low-carbohydrate diet 9 days before KMT initiation, mild ketosis (0.5 mmol/L beta hydroxybutyrate) was measured prior to structured dietary guidance. Therapeutic levels of nutritional ketosis (≥1.5 mmol/L beta hydroxybutyrate) were only achieved after implementing structured support with KMT. Testing compliance was 55% complete for daily ketone measures and 56% complete for daily glucose measures over the 25-week period. Blood glucose and beta-hydroxybutyrate level monitoring showed that nutritional ketosis was achieved at 1.5 mmol/L (Figure 1).

After 8 weeks of KMT, the patient reported mood instability after a period of marked improvement. In consultation with her KMT clinician, the possibility of potentiation effects was discussed. Following careful consideration with her prescriber, low-dose naltrexone was discontinued, resulting in symptom resolution.

The patient started self-initiated structured resistance training and sprint-based exercise at week 18. Follow-up weight and body composition measurements assessed using a Tanita® bioelectrical impedance scale approximately 26 weeks after KMT initiation recorded a weight of 128.0 lbs (58.1 kg), fat mass of 29.8 lbs (13.5 kg), and muscle mass of 93.2 lbs (42.3 kg).

### 2.3 Evaluation of intervention outcomes

Symptom severity was assessed using validated instruments administered at baseline and throughout the KMT intervention period. Baseline symptom severity was evaluated retrospectively at the first KMT session, 9 days after the patient independently initiated carbohydrate restriction. Because the patient already reported mild ketosis and subjective mood improvements at the time of baseline assessment, the symptom severity scores collected likely represent a conservative estimate of the patient’s initial symptom severity.

Quantitative assessment of symptom change was conducted using the PCL-5, Patient Health Questionnaire-9 (PHQ-9), Generalized Anxiety Disorder-7 (GAD-7), and Depression Anxiety Stress Scales-42 (DASS-42), which were administered at baseline and predetermined intervals through intervention completion at week 27. To provide a comprehensive evaluation of symptom remission at week 27, additional assessments were conducted using BDI and BAI.

Changes in symptom severity were quantitatively assessed using PCL-5, with scores ≥33 suggesting clinically significant symptom severity, and scores ≤10 indicating minimal symptoms and suggesting remission (52–54). Although the PCL-5 is validated for assessing symptom severity and monitoring symptom changes over time, it is not intended for diagnostic purposes (53), as clinical diagnosis of PTSD requires comprehensive clinical evaluation (55).

**Figure 2:**
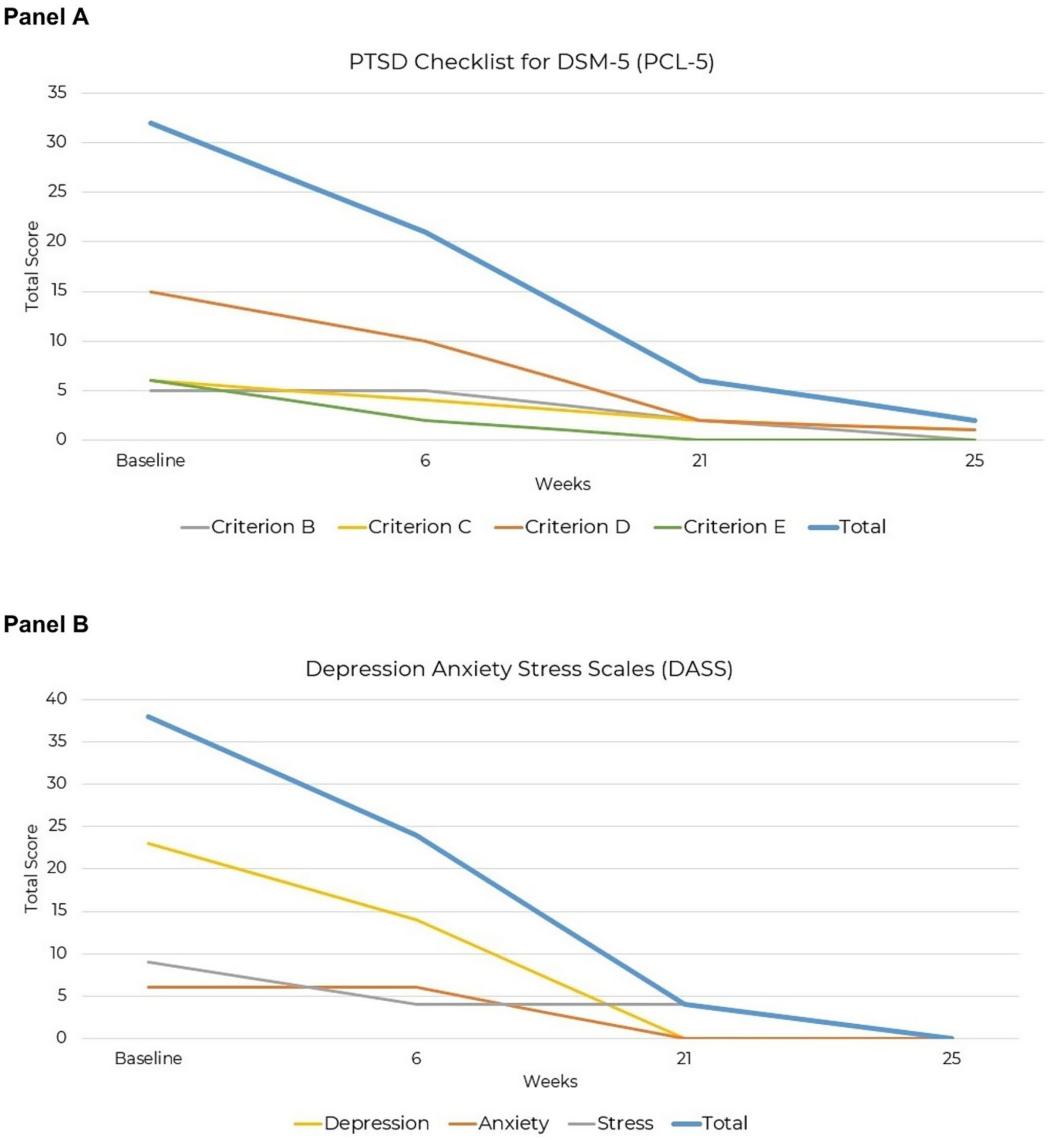
Reduction in PTSD symptoms severity over 25 weeks of KMT. **(A)** PCL-3. **(B)** DASS-42.

At the start of the intervention, the patient’s total PCL-5 score was 32, characterized by Criterion B intrusive symptoms severity of 5, Criterion C avoidance severity of 6, Criterion D negative alterations in cognition and mood severity of 15, and Criterion E alterations in arousal and reactivity severity of 6. By week six, the total score improved to 21, with reductions observed in Criterion C severity of 4, Criterion D severity of 10, and Criterion E severity of 2. Continued improvement was observed by week 21, with a total score of 6, reflecting minimal residual symptoms in Criterion B severity of 2, Criterion C severity of 2, Criterion D severity of 2, and resolution of Criterion E symptoms (severity of 0). At week 25, remission was confirmed with a total score of 2, indicating minimal residual symptoms in Criterion C severity of 1 and Criterion D severity of 1 (Figure 2A).

The DASS-42 is a validated self-report tool for assessing the severity of depression, anxiety, and stress symptoms, with scores categorized as normal, mild, moderate, severe, or extremely severe (56, 57). At the start of the intervention, the patient’s total DASS 42 score was 38, characterized by severe depressive symptoms with a score of 23, mild anxiety (score of 6), and moderate stress (score of 9). By week 6, the total score improved to 24, primarily due to reduced depressive symptoms to a score of 14, unchanged mild anxiety (score of 6), and improved stress symptoms (score of 4). By week 22, substantial symptom resolution was observed, with a total score of 4, reflecting resolved depression and anxiety with minimal residual stress. At week 25, complete symptom resolution was sustained, with a total score of 0 across all three subscales (Figure 2B).

**Figure 3:**
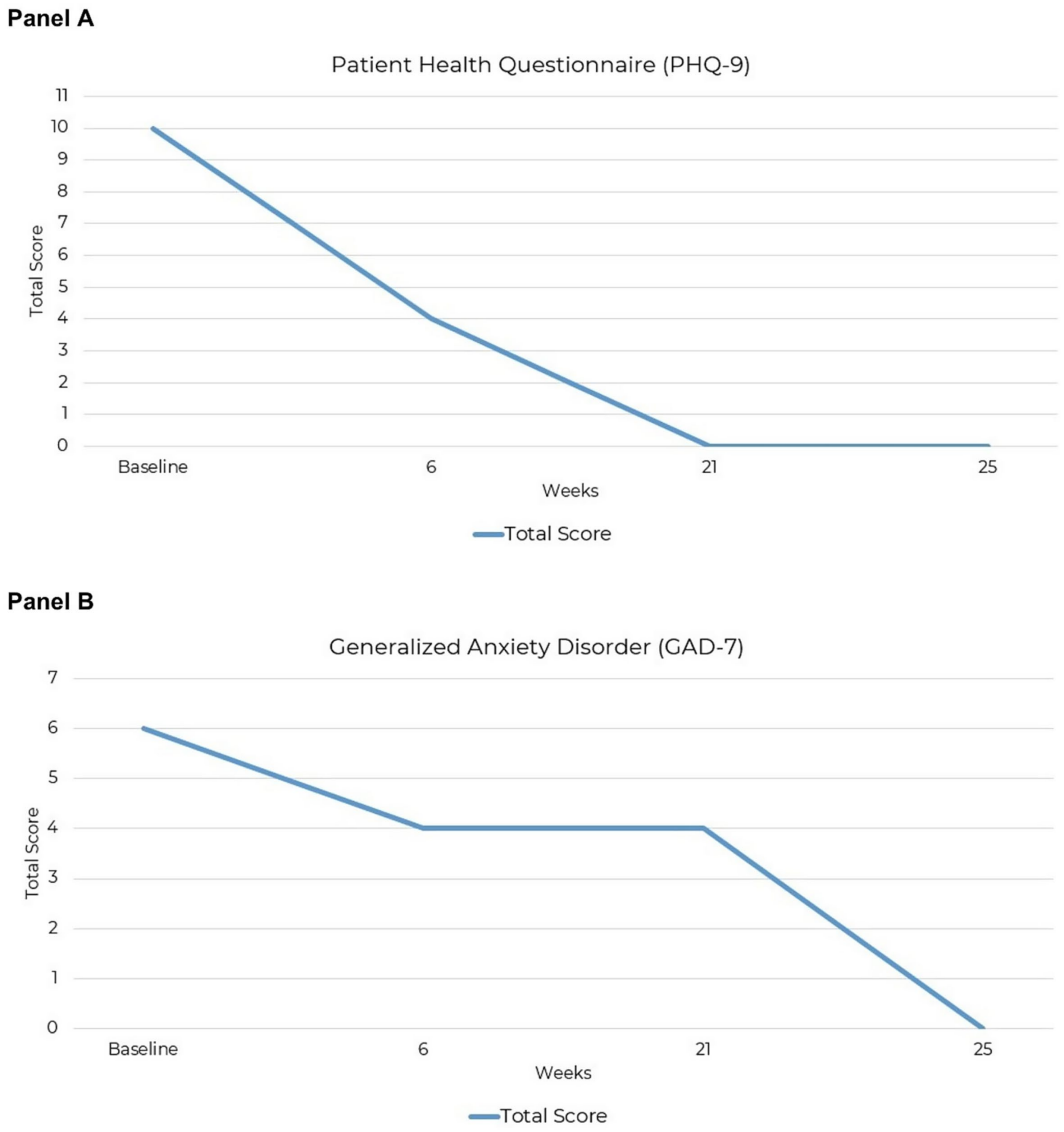
Reductions in the severity of PTSD symptoms over 25 weeks of KMT: **(A)** PHQ-9 and **(B)** GAD-7.

The PHQ-9 is a validated instrument used to measure the severity of depressive symptoms, with clinical scoring cutoffs categorizing severity as minimal (scores of 1–4), mild (5–9), moderate (10–14), moderately severe (15–19), and severe (20–27) (58). At the start of the intervention, the patient’s total PHQ-9 score was 10, corresponding to moderate depressive symptoms. By week 6, the total score decreased to 4, indicating a clinically meaningful improvement to minimal depressive symptoms. Full symptom resolution was observed by week 21, with a score of 0. The final assessment at week 25 confirmed remission of depressive symptoms, with a total score of 0 (Figure 3A).

The GAD-7 is a validated anxiety screening tool, although recent evaluations indicate limited reliability depending on the severity and clinical population (59). Using established clinical cutoffs, anxiety can be categorized as minimal (0–4), mild (5–9), moderate (10–14), and severe (15–21) (60). At the start of the intervention, the patient’s total GAD-7 score was 6, indicating mild anxiety symptoms. By week 6, the score decreased to 4, reflecting improvement to minimal anxiety symptoms. Symptoms remained minimal at week 21, with a score of 4, and fully resolved by week 25, with a score of 0 (Figure 3B).

Final assessment scores at week 25 indicated substantial reductions in psychiatric symptom severity. The PCL-5 score decreased from 64 at baseline to 2, meeting DSM-5 criteria for remission. Although BDI and BAI scores were not obtained at baseline, week 25 follow-up assessments showed notable improvements compared to historical data, with BDI scores decreasing from 39 to 1 and BAI scores decreasing from 45 to 1. These findings reflect meaningful clinical improvement and symptom remission after implementation with KMT.

### 2.4 Qualitative analysis

The patient reported improvements in mood, cognitive function, and overall wellbeing following KMT. Before treatment, she experienced severe, treatment-resistant depression and persistent suicidality despite multiple interventions.

*"I was SO depressed. I tried everything that I could think of and that anyone else could point me to. I felt as though I would never be happy."*

She described experiencing persistent suicidal ideation while concealing the severity of her distress.

*"I went on a trip and was terrified to go due to my extreme depression and suicidal ideation. I opened up a bit to my husband about being scared that I was going to die but I did not tell him that I wanted to drown in the ocean and felt that I might do that while I was there."*

She described a fundamental shift in her psychiatric state following KMT.

*"I wrote to my friends that I felt like my depression was gone."*

Observable improvements included increased psychological resilience, re-engagement in social relationships, enhanced emotional stability, and deeper connections with family members.

*"My relationship with my husband is better than it has ever been in 27 years. I feel great about my kids' independence while still having a close, deep relationship with them."*

Her ability to reflect on past trauma without emotional distress suggested an improvement in cognitive reappraisal and metacognitive awareness.

*"I still think of some of my traumas but they no longer have ownership over me. The memories come up and I am able to offer compassion to the woman I was when I experienced them."*

She reported a complete shift in both self-perception and future outlook:

*"For the first time in a LONG time I feel genuinely happy… I have energy, tenacity, focus, goals, and confidence. I am strong and healthy. I feel alive and, more importantly, grateful to be alive."*

These qualitative data extend beyond symptom severity measures, describing improvements in psychological resilience, cognitive reappraisal, social relationships, self-perception, and overall quality of life. These direct quotes offer insight into meaningful aspects of recovery for this single case report not fully captured by the quantitative symptom-based measures. Qualitative methods are increasingly recognized as essential to fully understanding patient experiences and meaningful recovery in psychiatric contexts (61–63).

## 3 Discussion

This case report contributes to limited evidence on the efficacy of KMT for treatment-resistant PTSD. It provides comprehensive documentation of symptom remission through validated psychometric assessments and qualitative data collection. Adjustments such as medication discontinuation based on clinical judgment demonstrate the adaptability necessary for effective metabolic psychiatry interventions. Residential group program treatment failure may reflect documented challenges for individuals with MST-related PTSD in tolerating and engaging with group-based treatment formats (64–66). Limitations include the absence of baseline mood assessments prior to dietary carbohydrate restriction, the inability to capture baseline dietary carbohydrate consumption, and the lack of comprehensive baseline measures of body composition. Alternative explanations for the observed symptom improvements, such as placebo effects, spontaneous remission, or the potential confounding impact of discontinuing low-dose naltrexone, cannot be ruled out. Potential interactions between KMT and naltrexone remain unknown, and future controlled studies investigating interactions with medications commonly used in psychiatry are needed. Additionally, the patient’s independent initiation and proactive engagement with KMT may reflect a high level of motivation, potentially enhancing treatment adherence and outcomes. Furthermore, long-term safety concerns regarding ketogenic diets in adult psychiatric populations, particularly cardiovascular and metabolic effects, are not yet clearly defined in existing literature and warrant investigation in future controlled studies.

Emerging evidence suggests that alterations in the gut microbiome, inflammation, and immune dysregulation may influence PTSD pathophysiology by modulating stress responses and neurotransmitter signaling along the microbiota–gut–brain axis (27, 28, 67). This is of clinical interest as these same pathways are modulated by KMT through documented effects on gut microbiota composition, inflammatory signaling, and immune regulation (68, 69). Emerging evidence from animal models and limited clinical data suggest that sex-specific hormonal factors might influence responses to ketogenic metabolic therapy, highlighting an important area for future controlled research (70–72). The findings of this report are consistent with and meaningfully extend previous preliminary case reports and feasibility studies of ketogenic dietary interventions for psychiatric disorders, including limited evidence specifically in PTSD (48–50).

Although outcomes from a single case have limited generalizability, the documented clinical improvements suggest KMT may have therapeutic potential for individuals with treatment-resistant PTSD and support the need for further controlled investigations. Future dedicated mechanistic reviews or experimental studies providing a comprehensive exploration of possible underlying biological mechanisms are warranted. This case expands existing metabolic psychiatry literature by demonstrating symptom remission in treatment- resistant PTSD following KMT. These results reinforce the potential of targeted dietary interventions to effectively address metabolic dysfunction contributing to persistent psychiatric symptoms.

## Data Availability

The datasets presented in this article are not readily available because of ethical and privacy restrictions. Requests to access the datasets should be directed to the corresponding author.
