# Supplementary material for: Ketogenic metabolic therapy for treatment-resistant post-traumatic stress disorder (PTSD): a retrospective case report
Source: Front Nutr. 2026 Feb 17;13:1755107. doi: 10.3389/fnut.2026.1755107 (PMC12953541; doi:10.3389/fnut.2026.1755107)
Supplement: Supplementary file 1 [file Table_1.DOCX]

## Supplementary Table 1: Historical psychiatric symptom severity prior to initiating ketogenic metabolic therapy

| **Measure** | **Total Score** | **Criterion B** | **Criterion C** | **Criterion D** | **Criterion E** |
| --- | --- | --- | --- | --- | --- |
| PCL-5 | 64 | 20 | 4 | 21 | 19 |
| BDI | 39 | - | - | - | - |
| BAI | 45 | - | - | - | - |
| **PCL-5** *(Posttraumatic Stress Disorder Checklist for DSM-5)*: A validated, self-report questionnaire assessing PTSD symptom severity across four DSM-5 symptom clusters. These include Criterion B (intrusive symptoms), Criterion C (avoidance), Criterion D (negative alterations in cognition and mood), and Criterion E (alterations in arousal and reactivity). | | | | | |
| **BDI** *(Beck Depression Inventory)*: A widely used self-report measure evaluating the severity of depressive symptoms. | | | | | |
| **BAI** *(Beck Anxiety Inventory)*: A validated self-report instrument assessing the severity of anxiety symptoms. | | | | | |

Scores reflect assessments completed approximately two years prior to initiating ketogenic metabolic therapy, indicating severe PTSD, depression, and anxiety symptoms, consistent with clinical diagnoses and limited response to previous interventions.
